# Optimization of CRT programming using non‐invasive electrocardiographic imaging to assess the acute electrical effects of multipoint pacing

**DOI:** 10.1002/joa3.12153

**Published:** 2019-01-14

**Authors:** Benjamin J. Sieniewicz, Tom Jackson, Simon Claridge, Helder Pereira, Justin Gould, Baldeep Sidhu, Bradley Porter, Steve Niederer, Cheng Yao, Christopher A. Rinaldi

**Affiliations:** ^1^ Division of Imaging Sciences and Biomedical Engineering King's College London London UK; ^2^ Cardiology Department Guys and St Thomas’ NHS Foundation Trust London UK; ^3^ CardioInsight Technologies, Medtronic Minneapolis Minnesota

**Keywords:** CRT, electrocardiographic mapping, multi‐point pacing, multi‐site pacing, non‐responders

## Abstract

**Aim:**

Quadripolar lead technology and multi‐point pacing (MPP) are important clinical adjuncts in cardiac resynchronization therapy (CRT) pacing aimed at reducing the rate of non‐response to therapy. Mixed results have been achieved using MPP and it is critical to identify which patients require this approach and how to configure their MPP stimulation, in order to achieve optimal electrical resynchronization.

**Methods & Results:**

We sought to investigate whether electrocardiographic imaging (ECGi), using the CARDIOINSIGHT ^™^ inverse ECG mapping system, could identify alterations in electrical resynchronization during different methods of device optimization. In no patient did a single form of programming optimization provide the best electrical response. The effects of utilizing MPP were idiosyncratic and highly patient specific. ECGi activation maps were clearly able to discern changes in bulk LV activation during differing MPP programming. In two of the five subjects, MPP resulted in more rapid activation of the left ventricle compared to standard CRT; however, in the remaining three patients, the use of MPP did not appear to acutely improve electrical resynchronization. Crucially, this cohort showed evidence of extensive LV scarring which was well visualized using both CMR and ECGi voltage mapping.

**Conclusions:**

Our work suggests a potential role for ECGi in the optimization of non‐responders to CRT, as it allows the fusion of activation maps and scar analysis above and beyond interrogation of the 12 lead ECG.

## 1 BACKGROUND

Cardiac resynchronization therapy (CRT) aims to restore regional activation synchrony and enhance cardiac contractility and the mechano‐energetic efficiency of the heart.^1^ Multi‐point pacing (MPP) has been developed as a tool to reduce the rate of non‐response.^2^, ^3^, ^4^ Intuitively, activating the heart from multiple locations could achieve more effective resynchronization; bypassing scarred myocardium and enabling the recruitment of a greater proportion of the left ventricle, resulting in increased conduction velocities and a reduction in the total activation time.^5^ Whilst some authors have shown improvements in acute hemodynamics^6^ and chronic echocardiographic remodeling,^2^, ^4^ recent data have suggested that its efficacy may be confined to a small proportion of patients and that its effect is conditional on specific electrical and anatomical^7^, ^8^ parameters and programming.^3^ We sought to investigate how myocardial activation varied during programming optimization and whether non‐invasive body surface mapping technology might be capable of identifying patients who may derive the most benefit from MPP.

## 2 METHODS

Patients on optimal medical therapy (OMT) meeting European Society of Cardiology (ESC)^9^ and/or Heart Rhythm Society (HRS)^10^ criteria for CRT implantation were enrolled into the study (Clinical Trails Number; NCT01831518, date approved 4 April 2013). The underlying aetiology of heart failure was determined using clinical history and cardiac MRI (CMR). Patients were implanted with a St Jude CRT Device (St. Jude Medical Inc., St. Paul, MN, USA) capable of MPP programmability and a Quartet^™^ quadripolar LV lead (St. Jude Medical Inc.). Initially devices were programmed according to the default manufacturer settings. The following day after implantation, each patient underwent an iterative CRT optimization procedure, guided by non‐invasive body surface mapping, looking to identify the optimal pacing settings.

### 2.1 Non‐invasive body surface mapping

A non‐invasive electrophysiological mapping study was performed using a high resolution electrocardiographic mapping system (ECVUE, CardioInsight Technologies Inc. Medtronic), as previously described.^11^ The patient's baseline presenting rhythm––either intrinsic sinus rhythm or RV paced rhythm––was first analysed using directional activation maps.^12^ Further mapping was undertaken using nominal CRT programming, before echo guided device optimisation was attempted. Finally, both local and extended bipolar MPP was used to further optimize the delivery of biventricular pacing. The ECSYNC software calculates four parameters assessing electrical activation:

1. Global Right/Left Ventricular Electrical Synchrony (VVsync): the mean activation time in the right ventricle minus the mean activation time in the left ventricle. Previously described as ventricular electrical uncoupling.
2. Global Biventricular Total Activation Time (VVtat): a measurement of the total time required for both ventricles to activate. Previously described as VVTAT.
3. Global Left Ventricular Total Activation Time (LVtat): a measurement of the total time required for all portions of the left ventricle to activate. Previously described as LVTAT.
4. Global Left Ventricular Dispersion of Activation (LVdisp): a measure of the dispersion of the activation times in the left ventricular region of interest.

Given the primary objective of CRT is to restore regional activation synchrony, we defined the optimal activation pattern as that which achieved the most effective degree biventricular resynchronization whilst simultaneously minimizing both biventricular and LV activation times. Electrical synchrony is specifically assessed by VVsync, where a figure of 0 represents identical LV and RV activation time.^13^ As such, the optimal pacing figuration was that which achieved a VVsync approaching 0, whilst also minimizing LV and BiV activation times.

**Figure 1:**
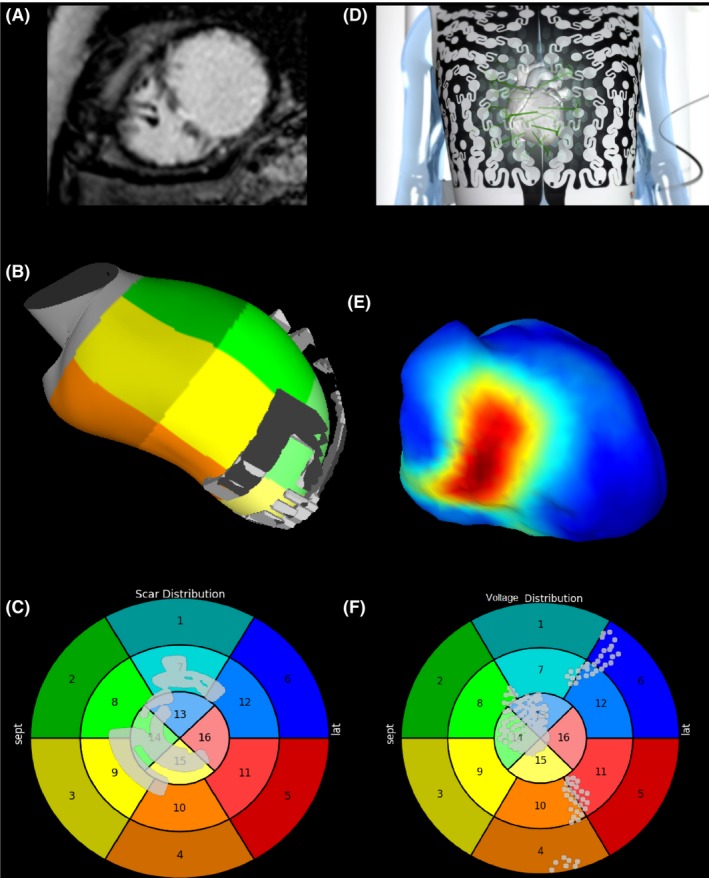
Multi‐panel plot showing a comparison between late gadolinium enhancement (LGE) derived scar from cardiac magnetic resonance (CMR) and areas of low voltage indicated scarred myocardium. A, LGE CMR in short axis showing areas of transmural hyperenhancement in the mid‐septum. B, Areas of LGE derived scar rendered onto on a 3‐D shell of the LV in RAO angulation. C, Areas of LGE displayed on a 16 segment bulls‐eye plot of the LV. D, The CARDIOINSIGHT ^™^ electrocardiographic mapping vest is applied to the thorax. E, Voltage thresholded CARDIOINSIGHT ^™^ electrocardiographic map in RAO angulation. Areas of <2mv are displayed in red. F, Areas of low voltage displayed on a 16 segment bulls‐eye plot of the LV

Epicardial voltage maps were also collected, in order to identify any areas of low voltage which may indicate areas of myocardial scar and fibrosis. Where possible, these were correlated against CMR data, see Figure 1.

### 2.2 Device optimisation

The patient's baseline presenting rhythm––either intrinsic sinus rhythm or RV paced rhythm––was first analyzed using directional activation maps.^12^ Subsequently, mapping was undertaken using nominal biventricular pacing settings (sensed atrioventricular [AV] delay 150 ms/paced AV delay 200 ms/LV & RV [VV] offset of 0 ms). Next, an echo guided iterative approach to device optimization was employed. The AV interval was optimized according to the maximal improvement in LV diastolic filling. An AV interval of 200 ms was first programmed followed by decrements of 20 ms until 60 ms.

The VV offset was optimized according to the maximal improvement in aortic pulsed‐wave Doppler velocity time integral, as previously described.^14^, ^15^ Pacing with the LV 60 ms ahead of the RV (+LV60) was initially programmed followed by +LV40, +LV30, +LV20, +LV15, simultaneous LV and RV pacing (sim), RV ahead by 20 ms (+RV20), +RV40, RV only pacing, and LV only pacing.

Once the optimal AV and VV intervals had been established and programmed, differing MPP settings were then acutely programmed. During each configuration, a non‐invasive electro‐anatomical mapping was obtained. The SJM CRT toolkit^™^ (St. Jude Medical Inc.) was used to identify the RV‐paced to LV‐sensed timings for each of the poles on the quadripolar lead. MPP was then programmed to pace the pole with the longest delay first and the pole with the shortest delay second. The right ventricular lead was always paced last. We delivered MPP with 5, 10, and 20 ms delays between each stimulus using both a local bipole configuration‐distal (D1) to mid 2 (M2) and proximal (P4), and an extended bipole configuration‐ D1 to RV coil and P4 to RV coil. This resulted in our testing 6 MPP settings per patient; 3 local and 3 extended bipole. In order to test the different MPP vectors for capture threshold and phrenic nerve stimulation, a number of standard biventricular recordings were also performed which served as comparators for individual patients.

## 3 RESULTS

### 3.1 Patient characteristics

A total of five patients were enrolled in the study.

### 3.2 Body surface mapping

The effect of changing AV delays, VV delays, the LV pacing vector, and finally the addition of MPP are shown in Table 1. In no patient did a single form of optimization provide the best electrical response. The mean electrical response using each strategy is shown in Table 1.

**Table 1:** Mean electrical response of each optimization strategy

	Electrical response			
	VVsync ms (range)	VVTAT ms (range)	LVTAT ms (range)	LV disp (range)
Optimization strategy				
AV optimization	−15.67 (−59 to 17)	89.03 (57‐129)	86.97 (57‐129)	28.33 (18‐43)
VV optimization	−4.9 (−28 to 42)	86.4 (51‐125)	81.3 (51‐125)	25.45 (15‐41)
Change in LV vector	−4.26 (−30 to 23)	84.62 (55‐144)	78.59 (55‐144)	24.72 (17‐48)
MPP on	−1.17 (−20 to 32)	87.6 (58‐141)	81.17 (54‐141)	25.03 (15‐48)

Epicardial voltage mapping showed the presence of scar in all of the ischemic patients which corresponded to LGE on MRI in the 3 cases where MRI was performed, see Figure 1. MPP had divergent effects on electrical activation in different patients that are described below.

### 3.3 Case 1

**Table:** 

Age	62
Sex	M
Aetiology	ICM
LVEF	28%
Rhythm	SR
QRS morphology	LBBB
QRS width	170
PR interval	210

Echocardiography demonstrated severe biventricular impairment and CMR demonstrated delayed sub‐endocardial late gadolinium enhancement consistent with prior infarction of the apex, antero/infero‐septum, and inferolateral wall. The epicardial voltage map was consistent with extensive scarring in the same distribution as the LGE visualized during CMR.

#### 3.3.1 Electrical effect of MPP

**Figure 2:**
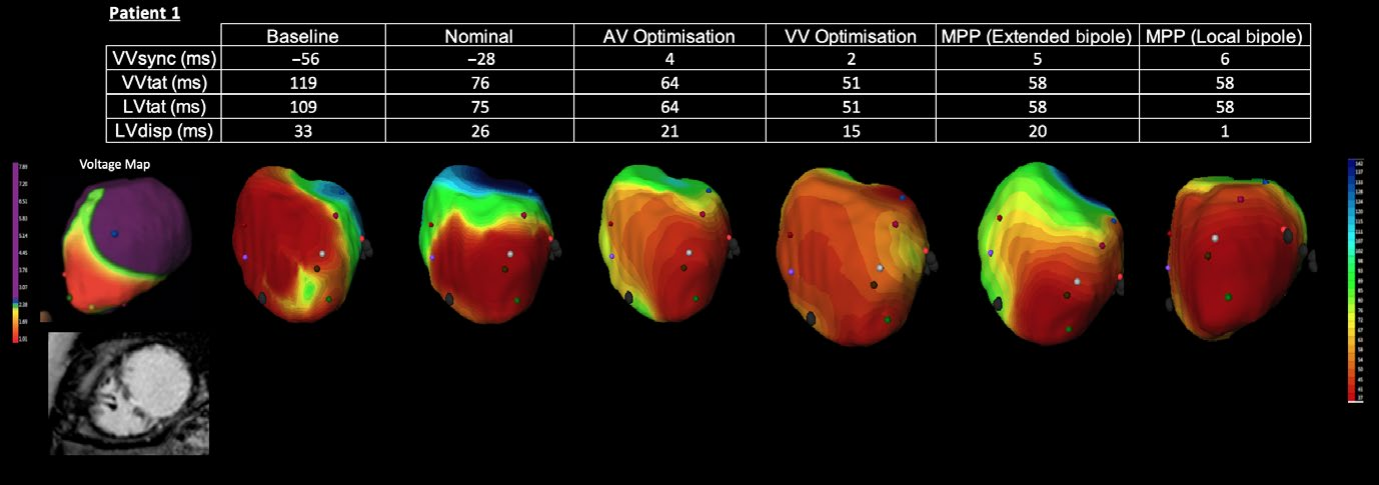
Electrocardiographic activation metrics and directional activation maps during device optimization of Patient 1. A voltage map thresholded to 2mv is shown with a still from the CMR short axis stack (SAX)

Intrinsic conduction exhibited the activation pattern typically observed in LBBB; ventricular activation is initiated at the distal branching of the right bundle, with activation of the left ventricular endocardium occurring after a significant delay, as a result of slow conduction through the interventricular septum (see Figure 2). This was associated with a broad QRS on the surface ECG. Conventional BiV pacing using nominal pacing settings yielded an improvement in activation pattern, with a significant reduction in both VVtat & LVtat as well as an improvement in VVsync. Further improvements in activation were observed following echo guided optimization of the AV and VV intervals. Activation maps undertaken during iterative programming optimization display advancement of the line of activation in comparison to nominal BiV activation. During extended bipolar pacing, with the RV coil as the anode, the activation maps appeared similar to conventional CRT, with apical to basal activation. Local bipolar activation achieved lateral to septal activation.

Whilst extended bipolar MPP programming was capable of reducing VVtat compared to nominal CRT, the use of local bipolar MPP did not lead to any further improvements in ventricular activation.

### 3.4 Case 2

**Table:** 

Age	50
Sex	M
Aetiology	ICM
LVEF	33%
Rhythm	SR
QRS morphology	LBBB
QRS width	176
PR interval	218

MRI demonstrated extensive thinning and scarring of the lateral wall. This was also displayed on the epicardial voltage map. CRT was performed with the LV lead inserted out of scar in an apical position.

#### 3.4.1 Electrical effect of MPP

**Figure 3:**
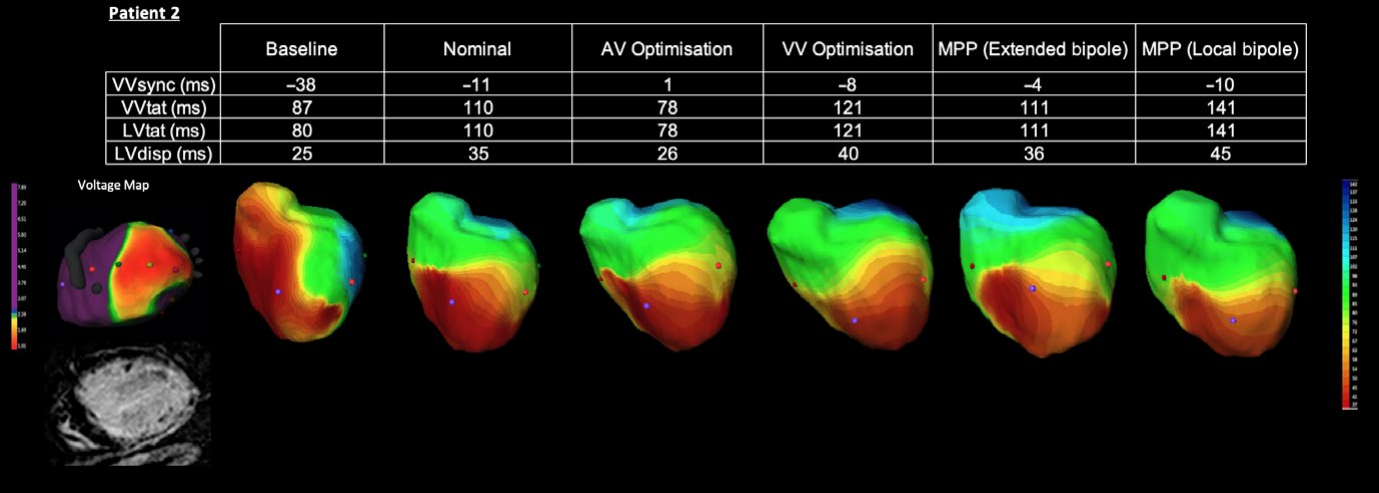
Electrocardiographic activation metrics and directional activation maps during device optimization of Patient 2. A voltage map thresholded to 2mv is shown with a still from the CMR short axis stack (SAX)

In this patient with extensive lateral scar, intrinsic activation was again characterized by typical LBBB propagation with delayed lateral LV wall activation (see Figure 3). Despite this patient having a broad QRS, nominal BiV pacing resulted in prolongation of the LVtat and VVtat, although improvements in V‐V synchronicity were observed. Attempts at optimizing CRT delivery though the use of both local and extended bipolar MPP proved equally ineffective and were associated with no significant change in activation pattern on the ECGi. Instead, iterative AV optimization proved the most effective optimization strategy, yielding a significant reduction in both LV and BiV activation times while also improving VVsync. This case demonstrates despite the deliberate avoidance of scar with an apical LV lead position, the use of MPP was unable to further optimize the pattern of activation.

### 3.5 Case 3

**Table:** 

Age	55
Sex	M
Aetiology	ICM
LVEF	13%
Rhythm	AF
QRS morphology	LBBB
QRS width	160
PR interval	N/A

This patient was unable to undergo CMR; however, the ECGi revealed an area of low voltage in the apical region in keeping with myocardial scar/fibrosis.

#### 3.5.1 Electrical effect of MPP

**Figure 4:**
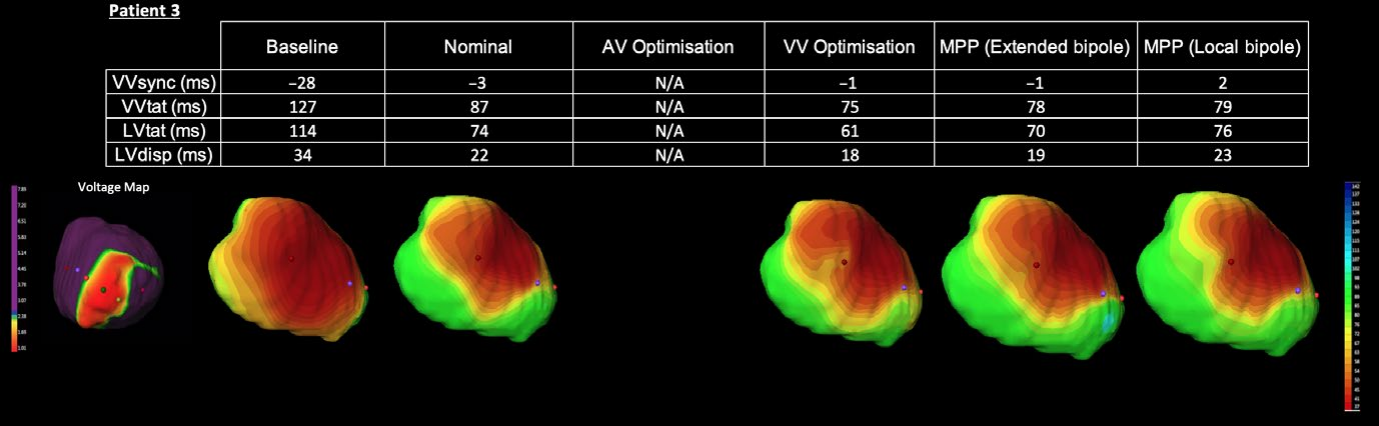
Electrocardiographic activation metrics and directional activation maps during device optimization of Patient 3. A voltage map thresholded to 2mv is shown

The pattern of LBBB activation with delayed activation in the lateral LV wall can again be observed on the baseline ECGi maps (see Figure 4). Interestingly this patient also had the longest LV and BiV activation time of the entire cohort but the shortest VVsync, suggesting activation in both ventricles was retarded. Activation was clearly delayed in the apical region, denoted by the blue isochrones on the activation map. This area corresponded to the previously identified area of low voltage tissue and likely represents delayed activation occurring in a region of scar tissue. Both BiV and LV activation times were significantly reduced during nominal BiV CRT. Electrical resynchronization also improved dramatically. Given this patient's underlying atrial fibrillation, AV optimization was not attempted; however, a small improvement in resynchronization and activation parameters was observed following VV optimization. Again, the addition of both extended bipolar and local bipolar MPP was unable to achieve a superior degree of electrical resynchronization, with the directional activation maps revealing a near identical pattern.

### 3.6 Case 4

**Table:** 

Age	59
Sex	M
Aetiology	ICM
LVEF	30%
Rhythm	SR
QRS morphology	LBBB
QRS width	160
PR interval	172

MRI demonstrated full thickness infarct in the mid anterior wall and anterio/infero‐septum and apex, which corresponded to areas of low voltage on the ECGi voltage map, see Figure 1.

#### 3.6.1 Electrical effect of MPP

**Figure 5:**
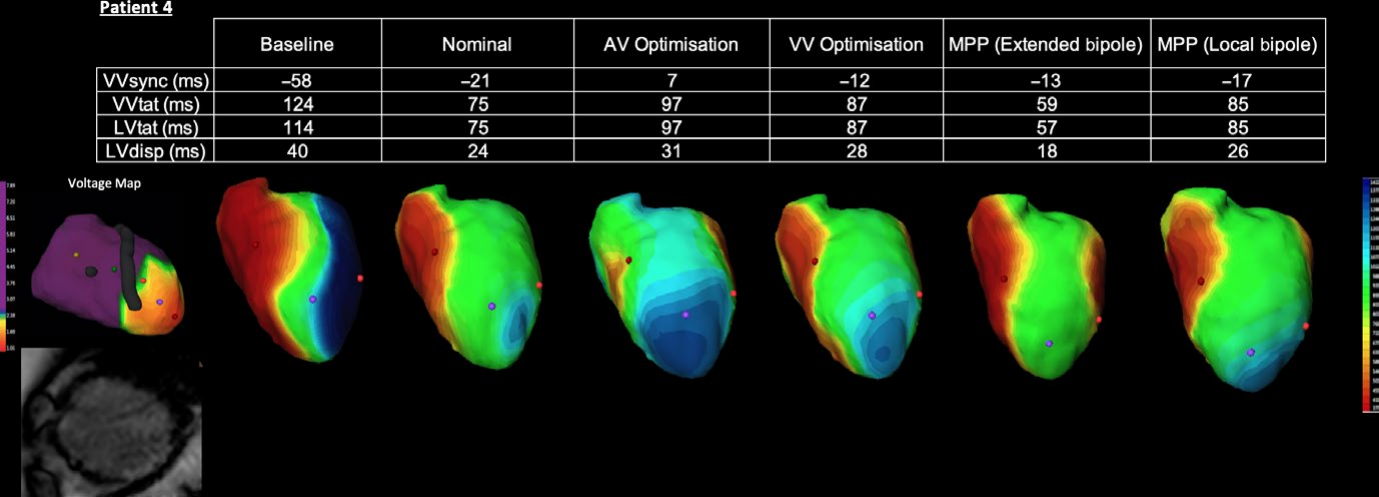
Electrocardiographic activation metrics and directional activation maps during device optimization of Patient 4. A voltage map thresholded to 2mv is shown with a still from the CMR short axis stack (SAX)

ECGi analysis of the intrinsic rhythm confirms activation is again delayed in the lateral LV wall (see Figure 5). The apical region also shows persistent delayed LV activation, which is in keeping with the CMR and voltage maps findings suggestive of apical scarring. Nominal BiV CRT achieved an improvement in electrical activation with reductions in both LVtat and VVtat and greater electrical resynchronization. In this case, AV optimization, VV optimization, and local bipolar MPP programming do not appear to confer any benefit over nominal BiV CRT. However, optimal vector selection using extended bipolar MPP results in more rapid activation of the left ventricle as demonstrated by the larger area of depolarized myocardium and reduction in delayed activation (blue) at the apex on the ECGi maps. These changes were associated with significant reductions in both LV and BiV activation time. In this example, Extended Bipolar MPP appears to offer a superior degree of resynchronization to conventional CRT.

### 3.7 Case 5

**Table:** 

Age	83
Sex	F
Aetiology	NICM
LVEF	35%
Rhythm	AF
QRS morphology	RV paced
QRS width	174
PR interval	N/A

The patient had long‐standing AF and had undergone implantation of a VVI pacing system in conjunction with an AV junction ablation.

#### 3.7.1 Electrical effect of MPP

**Figure 6:**
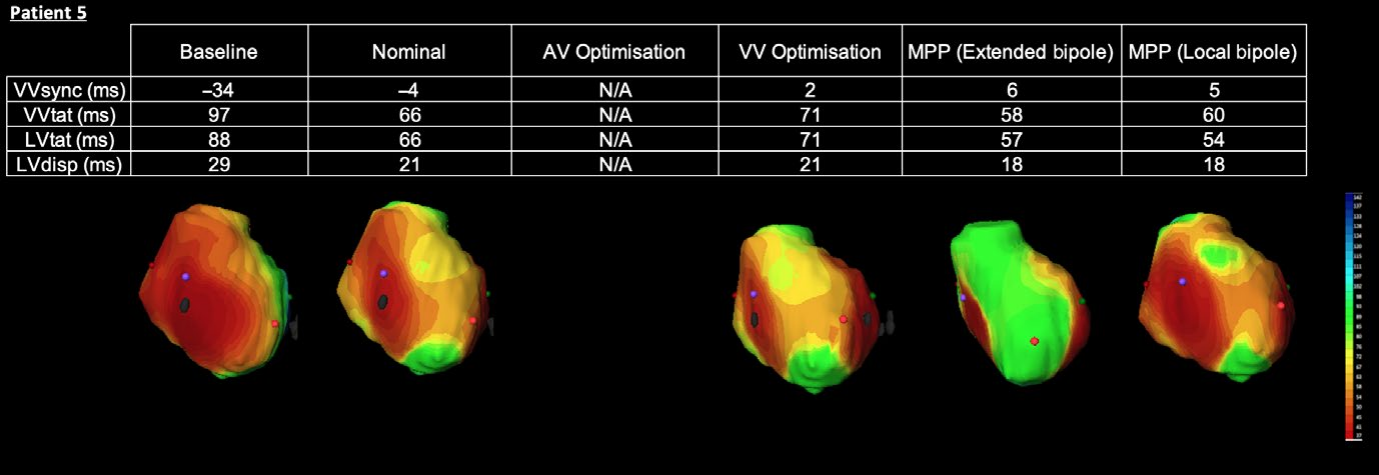
Electrocardiographic activation metrics and directional activation maps during device optimization of Patient 5

Baseline activation in this case demonstrates the pattern of activation typically associated with RV apical pacing (see Figure 6). The dark blue isochrones on the directional activation map denote an area of late activation in the posterolateral wall. Conventional CRT pacing with nominal settings achieves a dramatic improvement. Biventricular electrical resynchronization is almost entirely restored and both LV and BiV activation times are shorted. ECGi mapping now shows a widespread area of early activation occurring in the previously delayed posterolateral area. This is consistent with LV activation from a LV lead placed in a posterolateral tributary of the coronary sinus. Due to the patients underlying AF, no AV optimization has been attempted; however, VV optimization in this patient confers no obvious advantage. Both extended Bipolar MPP and Local Bipolar MPP result in a much larger area of early myocardial activation and an ensuing reduction in the LV and BiV activation times is apparent. In this case, the ability of MPP to more rapidly capture a greater area of the ventricle could potentially lead to further improvement and greater remodeling.

## 4 DISCUSSION

Our series demonstrates that non‐invasive mapping technology is able to accurately delineate the divergent electrical effects of programming optimization. Evaluation of the 12 lead ECG alone during biventricular pacing is frequently challenging due to the merging wave fronts^16^ and provides only a general overview of ventricular electrical activation.^11^ ECGi activation maps were clearly able to discern changes in bulk LV activation during differing MPP programming. In addition, voltage mapping was able to delineate areas of fibrotic tissue with showed good correlation with areas of scar defined using LGE CMR. Some degree of discrepancy between these modalities was expected as CMR can struggle to detect areas of homogenous microscopic diffuse fibrosis due to the low resolution of the image. In addition, ECGi is more sensitive at detecting zones of epicardial and transmural fibrosis but may not always be able to detect sub‐endocardial scar.

CRT delivered with nominal settings always proved superior to baseline activation and this improvement may explain why the majority of patients who receive CRT improve, without undergoing programming optimization. In three of our cohorts, the degree of biventricular resynchronization was further enhanced with iterative echo guided optimization of the AV and VV intervals. Whilst there is evidence to support this strategy,^14^, ^15^, ^17^ larger studies have failed to consistently prove it's efficacy.^18^, ^19^ Our results would suggest that optimization of the AV and VV intervals may indeed result in more effective resynchronization than can be achieved using nominal settings in a proportion of patients, but not all. This may go some way to explaining the equivocal data surrounding AV/VV programming optimization. Crucially, ECGi was able to detect subtle changes in activation during AV & VV programming optimization.

The effects of utilizing MPP were idiosyncratic and highly patient specific. In 2 of the 5 subjects (patients 4 and 5) Extended Bipolar and Local Bipolar MPP resulted in more rapid activation of the left ventricle compared to optimized echo optimized CRT. It is possible accelerating LV activation has the potential to improve the degree of cardiac resynchronization and as such, may explain the greater hemodynamic improvements^20^ and enhanced response rate^21^ observed during early studies of MPP stimulation.

The remaining 3 patients showed evidence of extensive LV scarring which was well visualized using both CMR & ECGi voltage mapping. The use of MPP in this cohort did not appear to acutely improve electrical resynchronization. In part, this may be explained by the focal nature of the scar burden,^22^ which can disrupt the efficacy of resynchronization pacing, especially when concentrated lateral or posterolateral walls.^23^ Our preliminary findings appear to confirm the hypothesis that MPP may be able to acutely improve electrical resynchronization in selected patients but the presence of extensive scar may preclude response irrespective of the stimulation strategy. Nevertheless, the Cardioinsight ^™^ ECGi system represents a non‐invasive technique capable of assessing the acute response to MPP and may be of use in identifying patients likely to gain the most from MPP as well as how best to configure this multi‐polar pacing.

## 5 LIMITATIONS

This is a small study and the results are hypothesis generating rather than conclusive. Our primary objective was to analyze how myocardial activation varied during device programming optimization and whether non‐invasive body surface mapping technology might be capable of identifying these changes in activation. Our hypothesis did not extend to evaluating rates of response to CRT and given the small number of patients, it would be impossible to draw reliable conclusions. Instead, non‐invasive electrical measurements were analyzed acutely and it is unclear whether these results can be extrapolated to the chronic delivery of CRT. Biventricular pacing which improves the degree of biventricular electrical resynchronization has been associated greater response.^11^, ^24^, ^25^, ^26^ However, non‐response to CRT is a multifactorial issue requiring a comprehensive assessment of the various pre‐implant, peri‐implant, and post implant factors.^27^ As such, improvement in clinical status is not a direct corollary of programming optimization even when this yields a superior degree of electrical resynchronization.

## 6 CONCLUSIONS

Iterative echo guided AV & VV optimization & quadripolar lead technology in conjunction with MPP are important clinical adjuncts to CRT pacing. The range of programming options provide greater cost efficiency by reducing the need for reintervention after implantation for technical issues including high capture thresholds, lead displacement, and phrenic nerve stimulation.^28^ Our analysis with ECGi mapping confirms that this tool is capable of detecting subtle changes in activation pattern achieved using different device optimization strategies. Furthermore, our findings suggest that traditional CRT with nominal settings is able to largely restore biventricular electrical synchronicity in selected patients and may explain the consistent response rate of 50%‐70% to conventional CRT.

Amongst carefully selected patients; however, the use of optimal device programming can achieve a superior degree of electrical resynchronization when compared to conventional CRT with nominal settings. However, these strategies are not without cost. Echo guided device optimization can be expensive and time consuming^29^ whilst MPP is associated with a reduction in battery longevity.^30^ Neither strategy has been consistently shown to be superior to conventional CRT with nominal settings in large multicenter studies.^7^, ^8^, ^18^, ^19^

Our analysis suggests that judicious use of device reprogramming optimization may be a useful strategy; however, the main issue remains identifying which patients may require this approach and then successfully optimizing their programming to achieve optimal electrical resynchronization. ECGi is a non‐invasive technique capable of accurately delineating the electrical effects of CRT pacing as well as the presence and distribution of myocardial scar. Our work suggests a potential a role for this tool in the optimization of non‐responders to CRT, as it allows the fusion of activation maps and scar analysis above and beyond interrogation of the 12 lead ECG.

## CONFLICTS OF INTERESTS

Authors declare no conflict of interests for this article.
